# Spectral performance evaluation of a second‐generation spectral detector CT

**DOI:** 10.1002/acm2.14300

**Published:** 2024-02-22

**Authors:** Leening P. Liu, Nadav Shapira, Sandra S. Halliburton, Sebastian Meyer, Amy Perkins, Harold I. Litt, Hans Ulrich Kauczor, Tim Leiner, Wolfram Stiller, Peter B. Noël

**Affiliations:** ^1^ Department of Radiology Perelman School of Medicine University of Pennsylvania Philadelphia Pennsylvania USA; ^2^ Department of Bioengineering University of Pennsylvania Philadelphia Pennsylvania USA; ^3^ Philips Healthcare Orange Village Ohio USA; ^4^ Diagnostic and Interventional Radiology (DIR) Heidelberg University Hospital Heidelberg Germany; ^5^ Department of Radiology Mayo Clinic Rochester Minnesota USA

**Keywords:** dual energy CT, spectral CT

## Abstract

**Purpose:**

The aim of this study was to characterize a second‐generation wide‐detector dual‐layer spectral computed tomography (CT) system for material quantification accuracy, acquisition parameter and patient size dependencies, and tissue characterization capabilities.

**Methods:**

A phantom with multiple tissue‐mimicking and material‐specific inserts was scanned with a dual‐layer spectral detector CT using different tube voltages, collimation widths, radiation dose levels, and size configurations. Accuracy of iodine density maps and virtual monoenergetic images (MonoE) were investigated. Additionally, differences between conventional and MonoE 70 keV images were calculated to evaluate acquisition parameter and patient size dependencies. To demonstrate material quantification and differentiation, liver‐mimicking inserts with adipose and iron were analyzed with a two‐base decomposition utilizing MonoE 50 and 150 keV, and root mean square error (RMSE) for adipose and iron content was reported.

**Results:**

Measured inserts exhibited quantitative accuracy across a wide range of MonoE levels. MonoE 70 keV images demonstrated reduced dependence compared to conventional images for phantom size (1 vs. 27 HU) and acquisition parameters, particularly tube voltage (4 vs. 37 HU). Iodine density quantification was successful with errors ranging from ‐0.58 to 0.44 mg/mL. Similarly, inserts with different amounts of adipose and iron were differentiated, and the small deviation in values within inserts corresponded to a RMSE of 3.49 ± 1.76% and 1.67 ± 0.84 mg/mL for adipose and iron content, respectively.

**Conclusion:**

The second‐generation dual‐layer CT enables acquisition of quantitatively accurate spectral data without compromises from differences in patient size and acquisition parameters.

## 1 INTRODUCTION

Computed Tomography (CT) is often one of the first imaging studies that a patient undergoes when entering the healthcare system and is part of the surveillance workflow for various pathologies and disease states. Traditionally, CT utilizes a polyenergetic x‐ray beam and energy‐integrating detectors to produce cross‐sectional image data based on attenuation or CT number measured in Hounsfield Units (HU). However, conventional CT still poses longstanding challenges for the clinical day‐to‐day routine. In certain situations, i.e. kidney stones^1^, ^2^ and liver lesions,^3^ different elements or tissues can be challenging to distinguish based only on CT number. Conventional CT is also not able to accurately measure physical quantities, for example, iodine density in units of mg/mL, because of beam hardening and other sources of error. Conventional CT image quality can additionally be negatively affected by patient habitus.

In contrast, spectral CT, as dual‐energy CT (DECT) or multi‐energy CT, enables differentiation and quantitative characterization of elements or tissues.^4^, ^5^ In particular, dual‐layer spectral detector CT allows routine acquisition of clinical DECT data without special acquisition protocols.^6^ It features a dual‐layer detector that separates low energy data from high energy data. The top layer detects low‐energy photons, while the bottom layer detects high‐energy photons. Projection data obtained simultaneously from both detector layers can then be utilized to generate spectral images in addition to conventional CT image data. This technology allows spectral data to be acquired on all studies for all patients without selecting any special “spectral protocols” beforehand.

For many organ systems, availability of spectral data frequently adds valuable information for CT interpretation.^7^, ^8^, ^9^, ^10^, ^11^, ^12^ The use of virtual monoenergetic images (MonoE) at low energies can improve contrast signal in vascular studies, including those with suboptimal enhancement.^13^, ^14^ MonoEs at high energies, on the other hand, can reduce artifacts, particularly those from implants and stents, to reveal information previously hidden by artifacts.^15^, ^16^, ^17^ Additionally, combining information from low and high energy MonoEs may help differentiate and quantify materials, such as iron overload in the liver that can be indicative of primary or secondary hemochromatosis.^18^ With accurate iodine measurements from iodine density images, perfusion deficits can be identified, thereby improving diagnostics for pulmonary embolism,^19^, ^20^ myocardial infarction,^21^, ^22^, ^23^ and oncological lesions,^24^, ^25^, ^26^, ^27^ for instance. In these applications and others, an increased z‐coverage with wider detectors has additional advantages including a reduction in acquisition time and larger anatomical coverage of the tissue(s) or organ(s) of interest per gantry rotation.

This study reports on the technical performance of a dual‐layer CT (Spectral CT 7500, Philips Healthcare) with a z‐coverage of 8 cm and an 80 cm bore. The system is equipped with a second‐generation dual‐layer detection system, which consists of a higher‐efficiency detector array coupled with a two‐dimensional anti‐scatter grid. In comparison, the previous generation detector array was a stick design, which read out the scintillator on one side and was limited by a one‐dimensional anti‐scatter grid. Our evaluation examines the spectral performance of the second‐generation system in regards to material quantification accuracy, tissue characterization capabilities, and acquisition parameters and patient size dependencies.

## 2 METHODS

### 2.1 Phantom

**FIGURE 1:**
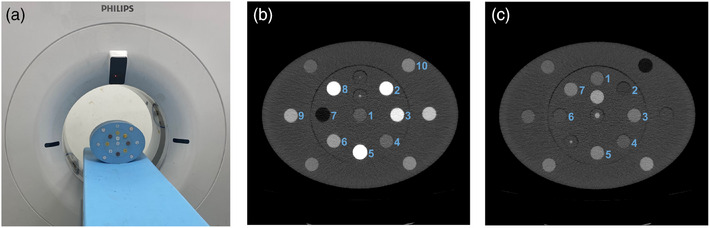
Insert configurations for the evaluation of accuracy of spectral results, quantitative stability, and material characterization. (a) The phantom was scanned on a second‐generation dual‐layer spectral detector CT. (b) For the assessment of accuracy and stability, relevant inserts included: (1) brain, (2) calcium 100 mg/mL, (3) iodine 10 mg/mL, (4) iodine 2 mg/mL, (5) calcium 300 mg/mL, (6) iodine 5 mg/mL, (7) adipose, (8) iodine 15 mg/mL, (9) blood + 4 mg/mL iodine, and (10) blood + 2 mg/mL iodine. (c) For demonstrating material separation with spectral results, inserts included: (1) liver + 10% adipose, (2) liver + 50% adipose, (3) liver + 5 mg/mL iron, (4) liver + 20% adipose, (5) liver + 11 mg/mL iron, (6) liver + 35% adipose, and (7) liver + 8 mg/mL iron.

A phantom with interchangeable material inserts (Multi‐energy CT Phantom, Gammex, Sun Nuclear) was utilized (Figure 1a). It consisted of a phantom extension (30 × 40 cm, "large") and an inner cylindrical portion with a diameter of 20 cm ("small"). The interchangeable cylindrical inserts included tissue‐mimicking inserts that are similar to human tissues in attenuation and elemental composition as well as inserts containing a known concentration of an element, for example, iodine. We calculated the expected attenuation at selected energies (keVs) as ground truth for comparison to measured values. These expected values were obtained by utilizing the elemental composition and physical density information supplied by the phantom manufacturer^28^ (Table 1):

(1) $$HU\;\left(E\right)=\left(\frac{\rho \sum_{i}w_{i}\times \mu_{i}\left(E\right)-\mu_{water}\left(E\right)}{\mu_{water}\left(E\right)}\right)\;\;\times 1000$$

where HU is the CT number of the insert at energy E, ρ is the physical density of the insert, $w_{i}$ is the mass fraction of element i, $\mu_{i}$ is the attenuation of element i at energy E, and $\mu_{water}$ is the attenuation of water at energy E. The expected range of values were then calculated by using the physical density tolerance prescribed by the manufacturer, 0.02 g/L. Additionally, the manufacturer provided values for adipose content and iron densities were utilized as ground truth for adipose and iron quantification.

**TABLE 1:** Elemental composition and physical density for phantom inserts.

	Elements [%]													
Insert	H	B	C	N	O	Na	Mg	Si	P	Cl	Ca	Fe	I	Physical density [g/L]
Adipose	9.73	0.05	71.41	2.71	14.35	0.18	0.00	1.11	0.00	0.12	0.34	0.00	0.00	0.961
Brain	8.23	0.04	65.75	2.05	19.70	0.15	1.23	0.94	0.00	0.13	1.79	0.00	0.00	1.053
Ca 100 mg/mL	6.33	0.00	57.24	2.41	25.74	0.00	0.00	0.00	0.00	0.10	8.18	0.00	0.00	1.244
Ca 300 mg/mL	4.03	0.00	40.80	1.54	33.81	0.00	0.00	0.00	0.00	0.07	19.76	0.00	0.00	1.551
Io 2 mg/mL	8.40	0.05	66.83	2.15	18.47	0.17	1.10	1.07	0.00	0.13	1.42	0.00	0.20	1.024
Io 5 mg/mL	8.37	0.05	66.68	2.14	18.39	0.17	1.10	1.07	0.00	0.13	1.42	0.00	0.50	1.029
Io 10 mg/mL	8.32	0.05	66.43	2.12	18.24	0.17	1.09	1.06	0.00	0.13	1.40	0.00	0.99	1.033
Io 15 mg/mL	8.27	0.05	66.18	2.11	18.09	0.17	1.08	1.05	0.00	0.13	1.39	0.00	1.48	1.038
Blood + 2 mg/mL Io	8.35	0.03	66.73	2.15	18.55	0.13	1.08	0.78	0.00	0.13	1.77	0.10	0.19	1.064
Blood + 4 mg/mL Io	8.33	0.03	66.64	2.15	18.49	0.12	1.08	0.78	0.00	0.13	1.76	0.10	0.38	1.064

For examining accuracy of spectral results and acquisition parameter and patient size dependence, a specific insert configuration (config 1, Figure 1b) was utilized. It included inserts for 2 mm iodine core, 5 mm iodine core, adipose, blood 40 HU, blood 70 HU, blood 100 HU, blood + 2 mg/mL iodine, blood + 4 mg/mL iodine, brain, calcium 50 mg/mL, calcium 100 mg/mL, calcium 300 mg/mL, iodine 2 mg/mL, iodine 5 mg/mL, iodine 10 mg/mL, and iodine 15 mg/mL. Specifically, select inserts were analyzed: brain, calcium 100 mg/mL, iodine 10 mg/mL, iodine 2 mg/mL, calcium 300 mg/mL, iodine 5 mg/mL, adipose, iodine 15 mg/mL, blood + 4 mg/mL iodine, and blood + 2 mg/mL iodine. For assessing material characterization, a different insert configuration (config 2, Figure 1c) was scanned and featured inserts for adipose, blood 40 HU, blood 70 HU, blood 100 HU, brain grey matter, brain white matter, iodine 5 mg/mL, liver + 10% adipose, liver + 20% adipose, liver + 35% adipose, liver + 50% adipose, liver + 5 mg/mL iron, liver + 8 mg/mL iron, liver + 11 mg/mL iron, and solid water.

### 2.2 Image acquisition

The phantom was scanned on a second‐generation dual‐layer spectral detector CT. Axial scans were performed at 100, 120, and 140 kVp for both phantom sizes (large, small). In addition, collimation width (16 × 0.625, 128 × 0.625 mm) and noise index/dose right index^29^ (DRI 10, 16, 23) were varied. The resulting tube current and volumetric CT dose index (CTDI_vol_, 32 cm) as a result of the selected dose right index for each combination of parameters is listed in Table 2. For some combinations, CTDI_vol_ was high but corresponded to acquisition parameter combinations that are not utilized clinically. Even so, all combinations were evaluated to ensure a comprehensive study. Scans were repeated three times for each insert configuration, phantom size, tube voltage, collimation width, and DRI level combination to capture the statistics of reconstructed results. Other relevant parameters are presented in Table 3. Spectral results were reconstructed for analysis, specifically iodine density maps, and MonoEs at 50, 60, 70, 100, 150, and 200 keV. Conventional CT images were also reconstructed to serve as a comparison to the MonoE 70 keV. Unlike other dual‐energy technologies, the dual‐layer technology is able to generate true conventional images by integrating data from the lower and upper layers.

**TABLE 2:** Corresponding radiation dose in terms of volumetric CT dose index for combinations of tube voltage, phantom size, and collimation.

			DRI 10		DRI 16		DRI 23	
Tube Voltage [kVp]	Size	Collimation [mm]	Exposure [mAs]	CTDI_vol_ [mGy]	Exposure [mAs]	CTDI_vol_ [mGy]	Exposure [mAs]	CTDI_vol_ [mGy]
100	large	16 × 0.625	106	7.8	167	12.2	270	19.8
100	large	128 × 0.625	106	5.1	167	8	270	13
100	small	16 × 0.625	25	1.8	39	2.9	107	7.8
100	small	128 × 0.625	25	1.2	39	1.9	107	5.1
120	large	16 × 0.625	64	7.6	101	11.9	270	31.9
120	large	128 × 0.625	64	5	101	7.8	270	20.9
120	small	16 × 0.625	16	1.9	25	3	69	8.1
120	small	128 × 0.625	16	1.2	25	1.9	69	5.3

Abbreviations: CTDI_vol_, volumetric CT dose index; DRI, dose right index.

**TABLE 3:** Scan acquisition and image reconstruction parameters.

Scanner model	Spectral CT 7500
Tube voltage	100, 120, 140 kVp
Rotation time	0.272 s
Collimation	16 × 0.625, 128 × 0.625 mm
Noise Index / DRI level	10, 16, 23
Slice thickness	2.5 mm
Iterative Reconstruction	iDose^4^ Level 3
Reconstruction filter	B
Reconstructed field of view	450 mm
Image matrix size	512 × 512
Pixel spacing (in *x* and *y*)	0.88 mm

### 2.3 Image analysis

An in‐house software tool^28^, ^30^, ^31^, ^32^ was used to automatically place the regions of interest (ROI) on each insert (covering 60% of the insert diameter, that is 17 mm) for MonoE 70 keV images of both insert configurations and phantom size combinations obtained with a DRI of 23. These ROI placements were then copied to corresponding locations on other spectral results to measure the mean and standard deviation of voxel values. Mean and standard deviation from ROI means of the three sets of images were calculated from multiple central slices from images of each repeated scan (4 slices x 3 scans at 16 × 0.625 mm collimation, 10 slices x 3 scans at 128 × 0.625 mm collimation). Measured values from each of the spectral results from config 1 were compared to the calculated expected range of values computed by applying well‐accepted material properties to the elemental composition and physical density values provided by the manufacturer. Values that fell within the expected range of values was considered accurate. These results were illustrated by plotting the CT number for each insert versus keV with an envelope that represents the range of expected values. The number of measurements that fell within the expected range of values were also reported. Deviation in iodine density results for iodine‐containing inserts in config 1 were visualized using a scatter plot where error bars corresponded to a single standard deviation of the means.

The stability of MonoE 70 keV relative to conventional images was investigated by determining patient size, tube voltage, radiation dose level, and collimation width dependencies using config 1 for each insert. Size dependence was calculated as the difference between measured values from the small and large phantom at a DRI of 16 for each individual insert. Tube voltage dependence, on the other hand, was calculated as the standard deviation between measured values at 100, 120, and 140 kVp at the moderate dose level while collimation dependence was defined as the difference between measured values at 16 × 0.625 and 128 × 0.625 mm. Size and tube voltage dependencies were additionally visualized in scatter plots to demonstrate differences in dependence between conventional and MonoE 70 keV images. Dose dependency was represented as the standard deviation between average mean values for three doses for each tube voltage, collimation, and size combinations. The same calculation was performed for average standard deviation (noise) values. Values were specifically shown for brain, iodine 10 mg/mL, and calcium 100 mg/mL inserts. Overall, for size, tube voltage, and dose dependency, the average and range of values across inserts were calculated to characterize general parameter dependency over a wide variation of inserts/materials.

To demonstrate material separability and characterization with MonoEs for iron and adipose, MonoE 50 keV values were plotted against MonoE 150 keV values for liver‐related inserts in config 2. Specifically, a low and high MonoE were chosen to represent the attenuation variation across the spectra, which provides the largest balance of spectral difference and noise. Accuracy of material quantification for the different acquisition parameters and phantom sizes was analyzed by first performing a linear two‐base material decomposition utilizing MonoE 50 and 150 keV data. This was achieved by linearly fitting CT number from inserts containing adipose and repeated for inserts containing iron to obtain an origin point that would theoretically correspond to a liver insert. A least squares fit was then applied to the data to obtain material unit vector representations for adipose and iron. Adipose content and iron density for each combination of acquisition parameters and phantom size was estimated from material basis projections for each insert. Both average error and root mean square error (RMSE) across the inserts for each set of acquisition parameters were calculated relative to specified manufacturer values of adipose content and iron density to evaluate material quantification accuracy.

### 2.4 Statistical analysis

All statistical analysis was performed in Python with scipy.stats. To compare the variance of MonoE values from each keV against conventional images across the variation of acquisition parameters and phantom size, Levene's test was utilized for each insert. A *p*‐value of 0.05 was considered significant. Additionally, because Shapiro‐Wilk test failed to reject normality, pairwise comparisons by insert were performed between conventional images and MonoE 70 keV with paired *t*‐test for phantom size, tube voltage, collimation, and dose dependence. A *p*‐value of 0.00625 was considered significant after applying Bonferroni correction for each metric.

## 3 RESULTS

### 3.1 Accuracy of spectral results

**FIGURE 2:**
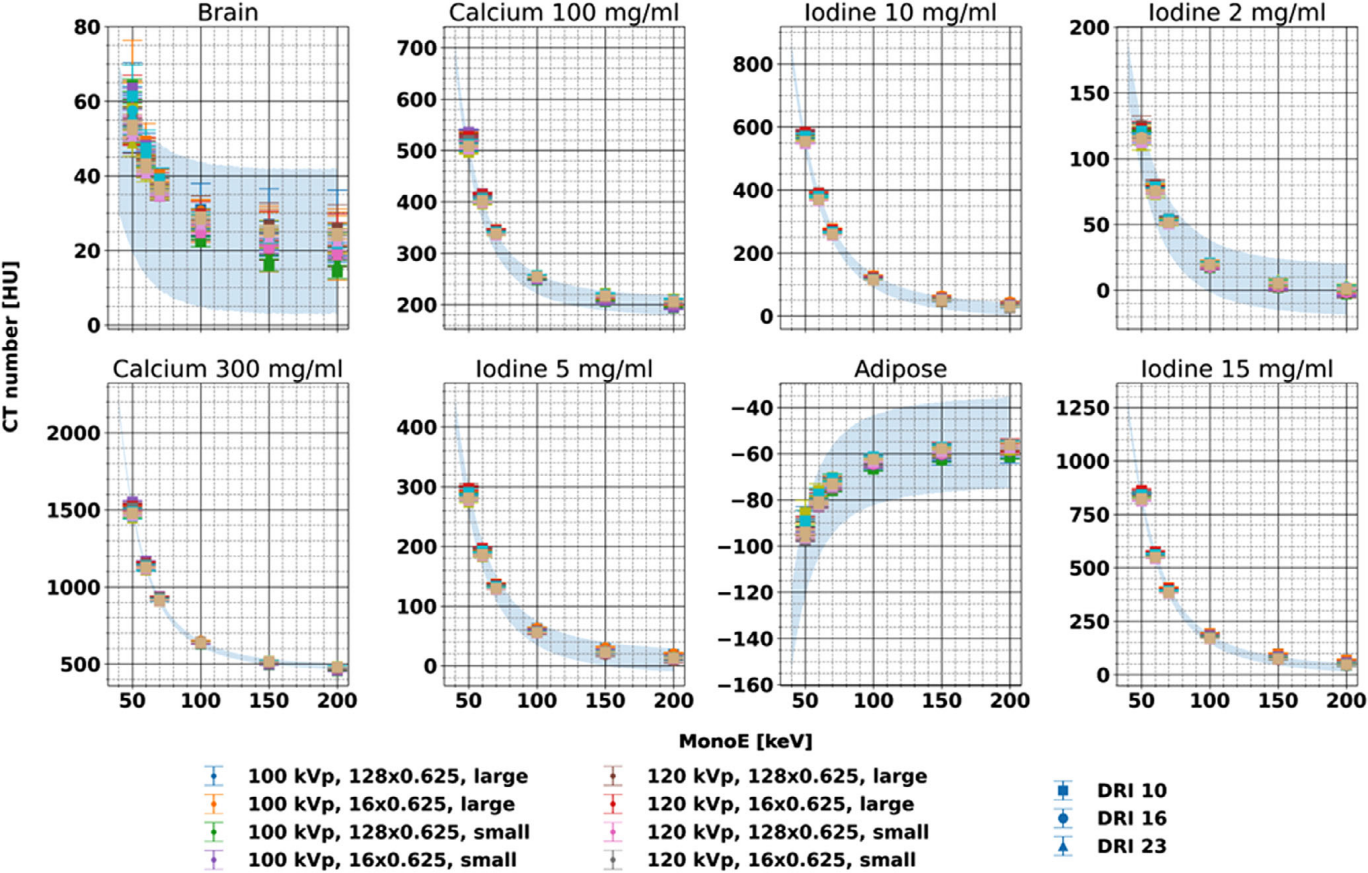
Accuracy of MonoE attenuation for each individual insert and acquisition parameter combination. Measured CT number for each insert and keV fell within the range of expected values (shaded blue area) calculated from elemental composition and manufacturer reported physical density, indicating accuracy. Please note different scaling of *y*‐axes. CT, Computed Tomography; MonoE, monoenergetic images.

**FIGURE 3:**
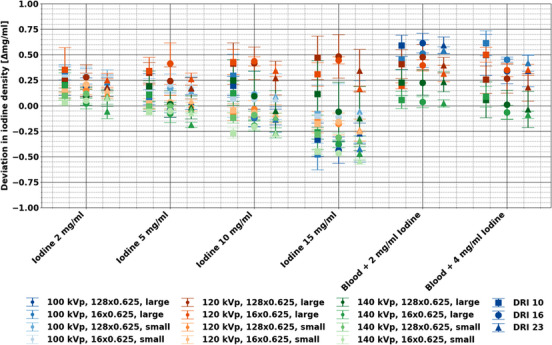
Comparison of relative error in measured iodine density for each iodine‐containing insert between scans with different phantom sizes, tube voltage, collimation, and dose levels. Measured iodine density was generally accurate and within ± 0.5 mg/mL, with larger deviations for inserts containing blood and iodine. The blood + 2 mg/mL and blood + 4 mg/mL inserts were only available with the large phantom size as the inserts were placed in the extension ring in config 1.

MonoEs and iodine maps demonstrated accuracy relative to expected values for insert measurements across all evaluated energies, phantom sizes, and acquisition parameters with respect to the expected range of values. Measured values for each insert fell within the expected range of values for 84.1% (1696/2016) of insert, tube voltage, collimation, dose, and MonoE combinations (Figure 2). The largest deviations from the expected attenuation value ranges corresponded to lower keV MonoEs with 15.6% (45/288) and 11.1% (32/288) not within range for MonoE 50 and 60 keV, respectively). High density materials (iodine, calcium) also resulted in values outside of the expected range, that is, 32.9% (83/252) and 25.4% (64/252) for calcium 100 and 300 mg/mL, respectively. Iodine density demonstrated good accuracy for iodine‐containing inserts with an error ranging from −0.58 to 0.44 mg/mL (Figure 3). For both MonoEs and iodine density maps, small differences in measured values were observed among different phantom sizes and acquisition parameters. The variations for MonoEs were significantly less than those observed with conventional images (*p*‐value range: 4.23 × 10^−15^–0.66), with the exception of the brain insert for MonoEs at all keVs except 70 keV.

### 3.2 Size and acquisition parameter dependence

**FIGURE 4:**
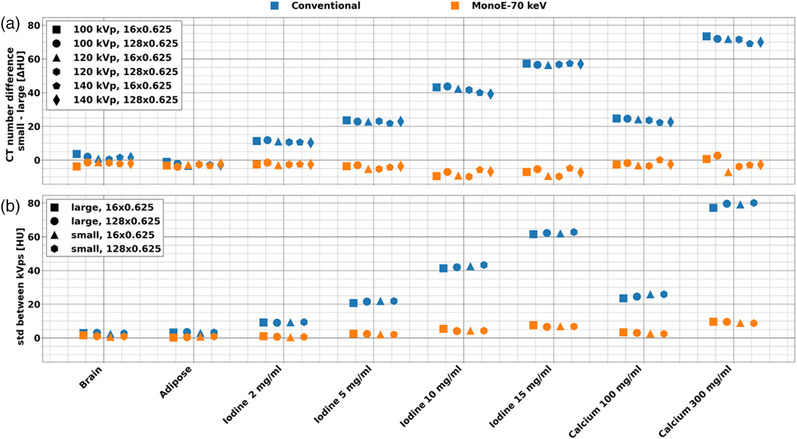
Size and tube voltage dependence of conventional and MonoE 70 keV images for each individual insert at DRI 16. The differences between the large and small phantoms (a) as well as tube voltages (b) were less evident in MonoE 70 keV compared to conventional images. This trend applied to all inserts and was particularly clear with higher density materials. DRI, dose right index; MonoE, monoenergetic images.

Size and tube voltage dependence of attenuation measurements were reduced for MonoE 70 keV in comparison to conventional images, while collimation width dependence maintained a similar low magnitude for both MonoE 70 keV and conventional images. Quantitative differences in conventional and monoE 70 keV images arose from differences in phantom size, ranging from −3.5 to 73.9 HU and −9.9 to 0.6 HU at 120 kVp for conventional and monoE 70 keV images, respectively, for the different inserts. These differences were significantly different between conventional and monoE 70 keV images for all inserts with the exception of brain and adipose insert (*p*‐value range: 2 × 10^−7^–8 × 10^−6^). At 100 kVp, size dependency ranged from −2.1 to 76.1 HU and −9.5 to 2.7 HU for conventional and MonoE 70 keV images, respectively (Figure 4a). Similarly, at 140 kVp, it averaged 27.07 and −1.16 HU for conventional and MonoE 70 keV, respectively. In addition, tube voltage dependence of insert attenuation averaged 37.1 and 4.5 HU for conventional and MonoE 70 keV images, respectively, with the small phantom while it averaged 37.9 and 4.0 HU for conventional and MonoE 70 keV images, respectively, with the large phantom (Figure 4b). The tube voltage dependence in conventional images corresponded to spectra differences, but these spectra differences did not affect quantification in MonoE 70 keV. Collimation dependence (10 vs. 80 mm), on the other hand, did not show a significant difference between conventional and MonoE 70 keV images for all inserts (*p*‐value range: 0.08–0.74). Quantitative differences for conventional and MonoE 70 keV images resulting from differences in collimation ranged from −4.6 to −1.0 HU and −5.8 to −0.7 HU, respectively. Dose dependence expressed in terms of average mean CT number (Table 4) and average standard deviation (noise) (Table 5) also did not demonstrate an overall significant difference between conventional and MonoE 70 keV images. Ranges for noise standard deviation with the large phantom were 14.5 and 11.7 HU for conventional and MonoE 70 keV images, respectively. With the small phantom, the ranges of noise standard deviation were 7.4 and 6.4 HU for conventional and MonoE 70 keV images, respectively.

**TABLE 4:** Average mean CT number for select inserts for different acquisition parameters.

Tube Voltage [kVp]	Size	Collimation [mm]	DRI	Average mean CT number [HU]					
				Conventional			MonoE 70 keV		
				Brain	Iodine 10 mg/mL	Calcium 100 mg/mL	Brain	Iodine 10 mg/mL	Calcium 100 mg/mL
100	Large	128 × 0.625	10	34.0	291.6	360.7	40.1	276.2	344.4
			16	36.4	293.2	358.1	38.5	272.5	342.8
			23	36.0	291.6	356.3	36.7	271.0	342.9
		16 × 0.625	10	37.2	295.0	363.6	40.3	276.8	345.6
			16	39.4	297.3	362.6	40.0	274.0	345.5
			23	37.7	296.0	360.5	38.7	273.2	346.3
	Small	128 × 0.625	10	37.6	334.8	385.4	36.3	266.7	341.7
			16	36.5	334.8	385.8	36.4	267.4	342.2
			23	36.7	335.0	385.6	35.4	267.0	341.2
		16 × 0.625	10	39.3	338.7	388.2	38.9	269.7	343.9
			16	38.7	338.7	388.2	38.4	269.8	343.8
			23	38.2	338.7	388.2	37.1	269.8	343.2
120	Large	128 × 0.625	10	32.4	232.4	325.5	36.8	270.5	343.6
			16	31.1	233.6	325.1	34.6	268.5	342.3
			23	31.7	232.1	322.7	35.2	265.9	340.6
		16 × 0.625	10	33.9	236.3	328.2	38.9	273.3	345.4
			16	34.3	236.7	327.9	37.8	269.9	344.7
			23	32.8	235.0	325.8	36.9	267.1	343.5
	Small	128 × 0.625	10	33.2	274.7	349.6	35.5	261.3	340.4
			16	32.4	274.6	349.3	35.2	261.1	340.1
			23	32.3	274.6	349.5	34.5	260.8	339.6
		16 × 0.625	10	34.3	277.9	351.8	37.2	263.4	342.0
			16	33.8	277.6	351.5	36.9	263.5	341.7
			23	33.6	277.8	351.7	36.1	263.3	341.2
140	Large	128 × 0.625	10	31.5	194.3	302.8	38.4	263.2	336.1
			16	32.1	195.2	302.8	35.9	259.6	335.6
			23	31.5	194.5	301.4	35.1	257.6	335.2
		16 × 0.625	10	30.3	191.5	301.3	39.2	266.3	340.6
			16	29.8	192.7	300.7	38.0	264.1	338.9
			23	30.0	191.8	299.7	37.7	261.9	338.2
	Small	128 × 0.625	10	33.0	233.5	325.4	36.2	257.4	336.2
			16	32.2	233.2	325.1	35.1	257.1	336.0
			23	32.5	233.2	324.7	34.8	256.3	335.5
		16 × 0.625	10	31.9	231.5	323.5	37.2	259.4	338.1
			16	30.9	231.1	322.8	36.4	259.5	338.1
			23	31.5	230.8	323.0	36.2	259.0	337.5

Abbreviations: CT, Computed Tomography; HU, Hounsfield Units.

**TABLE 5:** Average standard deviation of CT number for select inserts for different acquisition parameters.

				Average standard deviation of CT number [HU]					
				Conventional			MonoE 70 keV		
Tube Voltage [kVp]	Size	Collimation [mm]	DRI	Brain	Iodine 10 mg/mL	Calcium 100 mg/mL	Brain	Iodine 10 mg/mL	Calcium 100 mg/mL
100	Large	128 × 0.625	10	38.7	40.0	37.1	31.7	32.5	30.2
			16	33.6	32.9	29.9	27.9	26.9	24.9
			23	25.9	25.1	22.6	21.8	20.6	18.8
		16 × 0.625	10	40.3	37.6	35.4	32.8	30.6	29.0
			16	32.8	31.4	28.7	27.0	25.5	23.6
			23	26.1	24.7	22.8	21.9	20.5	18.9
	Small	128 × 0.625	10	17.2	14.5	14.9	14.6	11.9	12.3
			16	13.3	11.8	11.9	11.1	9.4	9.7
			23	8.0	7.3	7.7	6.7	5.5	5.8
		16 × 0.625	10	16.9	14.1	14.3	14.3	11.6	11.8
			16	13.2	11.3	11.4	11.1	9.2	9.4
			23	7.9	6.9	7.0	6.5	5.4	5.6
120	Large	128 × 0.625	10	34.0	32.5	31.0	28.4	27.2	26.1
			16	27.8	26.8	26.1	23.3	22.5	22.0
			23	16.3	16.2	16.0	13.9	13.9	13.5
		16 × 0.625	10	34.1	32.2	30.4	28.3	26.7	25.2
			16	28.9	26.8	24.9	24.2	22.4	20.8
			23	17.1	17.0	15.8	14.5	14.3	13.4
	Small	128 × 0.625	10	15.5	13.9	13.4	13.2	11.4	11.0
			16	12.2	11.2	11.5	10.2	8.8	9.5
			23	7.4	7.0	6.9	6.1	5.1	5.4
		16 × 0.625	10	15.3	13.0	13.3	12.9	10.7	11.0
			16	12.3	10.7	10.6	10.3	8.7	8.7
			23	7.4	6.5	6.5	6.1	5.0	5.2
140	Large	128 × 0.625	10	30.8	28.8	29.0	25.4	24.3	24.0
			16	23.8	22.5	21.7	19.9	18.8	18.4
			23	15.8	15.8	14.7	13.5	13.5	12.3
		16 × 0.625	10	30.7	28.1	27.6	25.6	23.4	23.0
			16	23.3	21.9	21.1	19.6	18.4	17.7
			23	15.6	15.6	14.7	13.2	13.2	12.4
	Small	128 × 0.625	10	15.0	14.3	13.9	12.7	11.6	11.4
			16	10.6	10.1	9.9	8.7	8.0	8.1
			23	7.2	6.8	7.0	5.8	5.2	5.4
		16 × 0.625	10	14.9	13.3	13.4	12.5	10.9	11.0
			16	10.3	9.6	9.5	8.6	7.7	7.8
			23	7.2	6.8	6.4	5.9	5.2	5.1

Abbreviations: CT, Computed Tomography; DRI, dose right index; HU, Hounsfield Units.

### 3.3 Material characterization

**FIGURE 5:**
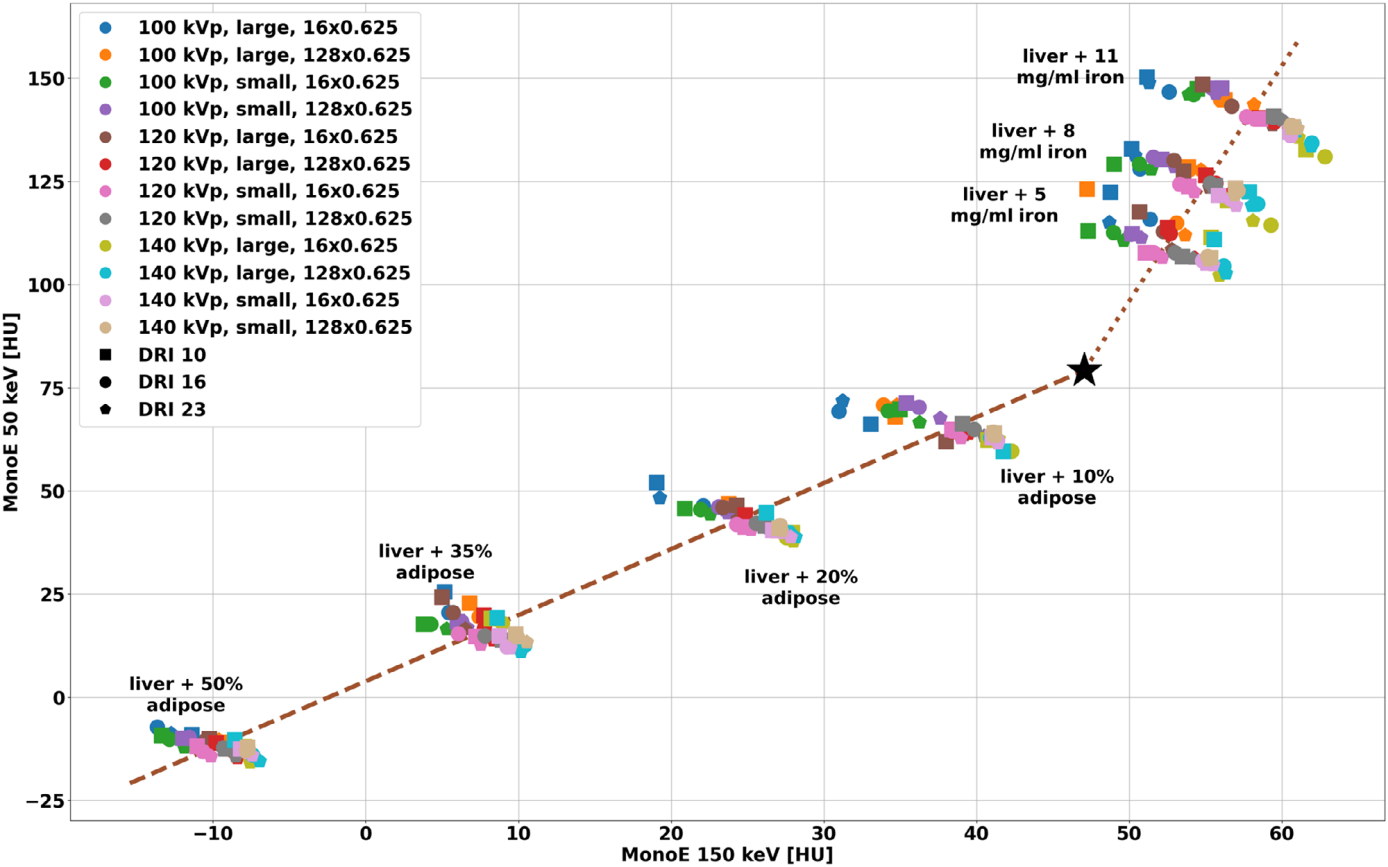
Separation of materials with MonoE 50 and 150 keV for config 2. CT numbers measured for MonoE 50 and 150 keV for the same insert exhibited small variations with different tube voltages, phantom size, collimation width, and dose levels. Values from the same insert aggregated and were distinct from other inserts. The origin (black star) and vectors for adipose content (brown dashed line) and iron density (brown dotted line) from the linear two‐base material decomposition are also shown. The results indicated the ability to quantify adipose content and iron concentration. CT, Computed Tomography; MonoE, monoenergetic images.

Figure 5 illustrates material characterization and quantification capabilities with MonoE 50 and 150 keV for inserts mimicking liver with different adipose content and iron concentration. With the reduced variations resulting from different phantom size and acquisition parameter combinations in MonoEs, values from the same insert aggregated around a single pair of MonoE 50 and 150 keV values but remained separated from values from inserts of different composition, showing excellent material characterization and separability. Moreover, both adipose‐containing and iron‐containing inserts demonstrated a linear relationship that corresponds to the material content. Based on specified manufacturer values, mean RMSE (Table 6) for adipose content and iron density across inserts averaged 3.49 ± 1.76% (range: 0.71%–5.18%) and 1.67 ± 0.84 mg/mL (range: 0.30–2.59 mg/mL) for acquisition parameter combinations, respectively, demonstrating material quantification accuracy. Additionally, average relative error across inserts ranged from −5.3 to 7.0% for adipose content and from −2.6 to 3.5 mg/mL for iron concentration (Table 6) for different acquisition parameters and phantom size combinations.

**TABLE 6:** Average relative error and root mean square error of adipose content and iron concentration quantification for different acquisition parameters.

				Average relative error		RMSE	
Tube Voltage [kVp]	Size	Collimation [mm]	DRI	Adipose [%]	Iron [mg/mL]	Adipose [%]	Iron [mg/mL]
100	Large	128 × 0.625	10	3.40	1.96	4.38	2.55
			16	2.30	1.37	2.98	1.57
			23	1.11	0.81	2.19	1.16
		16 × 0.625	10	7.02	3.53	7.35	3.73
			16	5.74	2.60	6.20	2.69
			23	6.85	3.05	7.29	3.26
	Small	128 × 0.625	10	3.51	1.80	3.67	1.90
			16	3.57	1.82	3.73	1.92
			23	2.20	1.08	2.42	1.23
		16 × 0.625	10	6.00	2.53	6.11	2.59
			16	5.12	2.17	5.18	2.22
			23	3.78	1.50	3.98	1.62
120	Large	128 × 0.625	10	0.13	0.30	0.71	0.67
			16	−1.00	−0.33	1.37	0.72
			23	−2.54	−1.25	2.69	1.32
		16 × 0.625	10	2.71	1.56	3.40	2.04
			16	1.92	1.09	2.35	1.29
			23	−0.27	−0.21	1.36	0.63
	Small	128 × 0.625	10	−1.49	−0.65	1.65	0.74
			16	−1.09	−0.48	1.33	0.53
			23	−2.47	−1.19	2.56	1.21
		16 × 0.625	10	0.41	−0.05	1.16	0.42
			16	0.81	0.09	1.21	0.30
			23	−0.49	−0.58	0.88	0.62
140	Large	128 × 0.625	10	−2.89	−1.00	3.44	1.47
			16	−4.70	−2.18	4.83	2.26
			23	−5.04	−2.46	5.14	2.53
		16 × 0.625	10	−3.23	−1.36	3.75	1.84
			16	−5.25	−2.58	5.74	2.93
			23	−4.79	−2.46	4.90	2.53
	Small	128 × 0.625	10	−3.33	−1.34	3.43	1.38
			16	−3.33	−1.33	3.38	1.35
			23	−3.72	−1.64	4.38	2.55
		16 × 0.625	10	−2.90	−1.37	2.98	1.57
			16	−3.49	−1.70	2.19	1.16
			23	−4.08	−2.03	7.35	3.73

Abbreviations: DRI, dose right index; RMSE, root mean square error.

## 4 DISCUSSION

In this study, our objective was to characterize the spectral imaging performance of a second‐generation dual‐layer spectral CT scanner. We demonstrated several benefits of spectral CT, including (i) a decoupling of quantitative values from acquisition parameters (100 vs. 120 kVp, 10 vs. 80 mm z‐coverage) and patient habitus, (ii) quantitatively accurate imaging, especially in the case of iodine, and (iii) material characterization capabilities, such as for iron.

Compared to first‐generation dual‐layer spectral CT, iodine quantification with the second‐generation dual‐layer spectral CT was improved. Utilizing the same multi‐energy CT phantom, prior studies with a first‐generation dual‐layer spectral CT demonstrated an iodine bias^33^ of 1.03 mg/mL and absolute error^34^ of 0.5 mg/mL at 120 kVp with the large phantom. Our study, however, demonstrated an improved iodine bias of 0.86 mg/mL and absolute error of 0.31 mg/mL at 120 kVp. Similarly, at 100 kVp, the iodine bias was small compared to first‐generation dual‐layer spectral CT, at −0.12 mg/mL, as was the absolute error at 0.23 mg/mL, highlighting improved iodine quantification. The previous system did not provide a 100 kVp scanning option. As a result of these improvements in iodine quantification with the second generation of systems, spectral CT may offer advantages over conventional oncological therapy assessment by using a biomarker that is based on iodine density. By utilizing iodine concentration measurements, one can obtain a functional image of perfusion patterns, which can be used to link biomarkers to changes in lesion shape, topology, texture, and vasculature^24^, ^25^, ^26^, ^27^ more accurately than the current oncological assessment criteria.^25^, ^35^, ^36^ Furthermore, by extending the maximum z‐coverage from 4 to 8 cm, iodine quantification in the myocardium may be used as a robust, semi‐quantitative proxy for myocardial perfusion, with iodine concentration serving as an indicator of blood distribution at a given time.^37^ To this end, we have presented results in this study which demonstrate that collimation width has minimal effect on iodine quantification. Additionally, spectral CT can be used to characterize materials outside the iodine domain, including iron.^38^, ^39^, ^40^ As spectral CT technology improves, as described in this study, it is becoming increasingly possible to characterize and detect iron content. This development may have clinical implications beyond liver imaging to include cardiac diagnostics as well.^41^

There are limitations to the present study. First, this study characterized the system by using technical phantoms and not clinical patient data. There will be a need to perform quantitative and qualitative disease‐specific studies in the future to determine performance across the diagnostic range. However, using phantoms, we have knowledge of ground‐truth material compositions and densities, which is not available in clinical trials. Additionally, despite the fact that the investigated system advances cardiac imaging with an extended z‐coverage, we did not investigate the effect of heart rate on spectral performance. In a follow‐up study, we intend to investigate the relationship between temporal resolution and spectral image quality. Finally, our study does not include direct comparisons with other spectral CT platforms. We and other investigators have described and compared the performance characteristics of other platforms, including photon‐counting, in previous publications.^31^, ^33^, ^37^, ^42^, ^43^

## 5 CONCLUSION

To conclude, we report the results of an experimental evaluation with respect to accuracy of spectral results, phantom size and acquisition parameter dependence, and material characterization of a second‐generation dual‐layer spectral CT. These results demonstrate improvements over first‐generation dual‐layer spectral CTs, which may improve the clinical value of spectral imaging and lead to more routine clinical adoption. As detector‐based spectral CT continues to demonstrate improved performance over conventional CT with its reduced dependence on acquisition parameters and its material characterization and quantification capabilities, its additional diagnostic benefits may become an expected part of routine clinical evaluation.

## AUTHOR CONTRIBUTIONS

Conception: Nadav Shapira, Sandra S. Halliburton, Sebastian Meyer, Amy Perkins, Harold I. Litt, Hans Ulrich Kauczor, Tim Leiner, Wolfram Stiller, Peter B. Noël. Design: Nadav Shapira, Sandra S. Halliburton, Sebastian Meyer, Amy Perkins, Harold I. Litt, Hans Ulrich Kauczor, Tim Leiner, Wolfram Stiller, Peter B. Noël. Data Acquisition: Sandra S. Halliburton. Analysis: Leening P. Liu, Nadav Shapira, Sebastian Meyer. Drafting: Leening P. Liu. Revising: Leening P. Liu, Nadav Shapira, Sandra S. Halliburton, Sebastian Meyer, Amy Perkins, Harold I. Litt, Hans Ulrich Kauczor, Tim Leiner, Wolfram Stiller, Peter B. Noël.

## CONFLICT OF INTEREST STATEMENT

Harold I. Litt and Peter B. Noël received a hardware grant from Philips Healthcare. Peter B. Noël receives research grant funding from Philips Healthcare. Tim Leiner, Peter B. Noël, and Wolfram Stiller are members of the CT Advisory Board of Philips Medical Systems. Hans‐Ulrich Kauczor receives research grant funding and speakers bureau fees from Philips Healthcare. Sandra Halliburton and Amy Perkins are employees of Philips Healthcare. The other authors have no relevant conflicts of interest to disclose.
